# Hydro-geometrical data analyses of River Atuwara at Ado-Odo/Otta, Ogun State

**DOI:** 10.1016/j.dib.2018.04.071

**Published:** 2018-04-25

**Authors:** Adebanji S. Ogbiye, Olumuyiwa O. Onakunle, David O. Omole

**Affiliations:** a Department of Civil Engineering, Covenant University, Ota, Ogun State, Nigeria; b Department of Civil and Environmental Engineering, University of Lagos, Akoka, Yaba, Lagos, Nigeria

**Keywords:** Atuwara River, Hydro-geometry, Water transport, Regression analysis, Particle transport modelling

## Abstract

The dataset analyzed in this article contains spatial and temporal values of the hydro-geometric parameters of River Atuwara. The hydro-geometrical data analyses of various sampling point on River Atuwara was examined and their geometric properties were taken with the use of a paddled boat, depth meter and global positioning system (GPS). The co-ordinates, width, depth, slopes, area, velocity, flow were gotten in-situ while the area and wetted perimeter were computed ex-situ. The statistical relationships between separate variables were considered using scatter plots and regression line equations. Inferences drawn from various variable comparisons can be used to validate predictive models for various time seasons.

**Specifications table**

**Table:** 

Subject area	*River Engineering, Water quality modelling*
More specific subject area	*Water transport modeling and simulation*
Type of data	*Table, image, text file, graph, figure*
How data was acquired	The referenced sampling points of the Atuwara river were taken with paddled boat and a depth finder. A global positioning system (GPS) unit was utilized to determine the location of the referenced points within the River Atuwara.
Data format	*Raw and analyzed*
Experimental factors	The study assumes that an irregular channel cross-sections can be represented with hydraulically equivalent (that is, area to wetted perimeter remains the same) trapezoidal cross-sections. Also, the processed hydro-geometric data assumes the top-width of each cross-section were unchanged.
Experimental features	*Very brief experimental description*
Data source location	*River Atuwara; located in Ado-Odo/Otta local government in the Southwestern part of Nigeria*
Data accessibility	*All the data are present in the data article.*

**Value of the data**

The hydro-geometric data presented are suggestive for the following purposes;

• The data can be used to develop some numerical models that simulate and predict the transport and fate of organic pollutants in the environment [1], [2], [3], [4], [5], [6], [7].
• The dataset helps to describe the temporal and spatial behavior of pollutants and nutrients in the Atuwara River.
• These field observatory data can be used to validate predictive model for various hydrological seasons.
• The hydro-geometric data set can serve as an indicator to decision makers for consideration of current and futuristic water pollution controls.

## 1 Data

The dataset comprises of hydro-geometric analyses of selected sampling points on the River Atuwara, located in Ado-Odo/Otta, in southwest Nigeria. The hydro-geometric data was collected with the use of equipment such as depth meter, paddled boat, tape measure, and a global positioning system. Fig. 3 is illustrative of the hydro-geometric data collection process. Geometric values are shown in Table 1, with their respective unit standards. Relationships between various units of measurement were derived statistically and presented in Fig. 4, Fig. 5, Fig. 6.

**Table 1:** October, 2008 Atuwara Rivers field measured hydro-geometric parameters.

***S/No.***	***Coordinates***		***Way points***	***Relative distance to STA-Atuara upstream*****(km)**	***Station description***	***WIDTH (m)***		***DEPTH(m)***				***Sides slope***		***Area*****(m^2^)**	***Velocity*****(m s**^**−1**^**)**	***Flow*****(m**^**3**^** s**^**−1**^**)**	***Manning's***	***Wetted perimeter*****(m)**	***Oxygen reaeration***	***Dispersion***
	***Northings***	***Eastings***				***Top (B)***	***Bottom (B***_***o***_***)***	***Left***	***Mid***	***Right***	***Mean (H)***	***S***_***s1***_	***S***_***s2***_	***A***_***C***_	***U***	***Q***	***n***	***P***	***K***_***a***_	***E***
									***Stream***											
1	523883	745372	STA	0	Atuara Upstream	13.1	11.56	0.74	0.89	0.8	0.81	1.04	1.13	10.6	0.41	4.351	0.035	13.899	3.452	4.93
2			STB	0.19	Abattoir	4.3	2.85	0.71	0.77	0.74	0.74	1	1.05	3.18	0.43	1.368	0.035	4.956	4.048	4.722
3			STC	0.24	Abattoir Downstream	8.4	6.74	0.86	1.26	0.8	0.97	1.21	1.13	7.58	0.43	3.25	0.035	9.598	2.698	5.743
4			STD	1.21	Sona Upstream	16.2	14.23	0.89	2.25	1.08	1.41	1.26	1.53	22.84	0.42	9.594	0.035	18.594	1.521	8.79
5			STE	1.26	Sona Discharge	8.6	6.51	1.14	2.03	0.95	1.37	1.61	1.34	11.78	0.38	4.477	0.035	10.821	1.511	7.727
6			STF	2.78	Ewupe Upstream	10.4	7.96	0.99	2.53	1.45	1.66	1.4	2.05	17.26	0.38	6.56	0.035	13.441	1.133	9.362
7			STG	2.83	Ewupe Discharge	13.4	10.81	1.02	2.68	1.57	1.76	1.44	2.22	23.58	0.4	9.434	0.035	16.732	1.065	10.45
8			STH	3.08	Ewupe Downstream	13.5	11.56	1.02	2.59	0.92	1.51	1.44	1.3	20.38	0.39	7.95	0.004	16.209	1.323	8.741
9			STJ	4.67	Afara Meje	16.9	15.26	1.32	2.17	2.96	2.15	1.87	4.19	36.34	0.41	14.897	0.004	23.892	0.798	13.083
10			STK	7.94	Ekusere	11.8	8.23	2.34	2.71	1.23	2.09	3.31	1.74	24.66	0.34	8.385	0.004	16.078	0.758	10.547
11			STL	8.36	Ekusere Down Stream	8.9	6.53	1.51	2.56	0.86	1.64	2.13	1.22	14.6	0.36	5.255	0.004	11.895	1.123	8.764
12			STM	9.28	Igboloye Upstream	9.4	6.1	1.79	2.22	1.51	1.84	2.53	2.13	17.3	0.32	5.535	0.004	12.815	0.891	8.74
13			STP	9.88	Igboloye Discharge	10.2	6.87	2.25	2.06	1.08	1.8	3.18	1.53	18.38	0.28	5.141	0.004	13.464	0.861	7.481
14			STQ	9.88	Igboloye 100 m Down Stream	12.3	9.62	1.94	2.19	0.74	1.62	2.74	1.05	19.93	0.31	6.177	0.004	15.133	1.061	7.454
15			STR	10.71	Igboloye 600 m Downstream	11.2	7.35	3.45	1.79	0.4	1.88	4.88	0.57	21.06	0.29	6.106	0.004	14.607	0.821	8.092
16	516392	738497	STS	10.81	Iju Water Works	16.9	10.46	5.02	3.11	1.39	3.17	7.09	1.96	15.62	0.32	4.995	0.004	25.362	0.394	4.387

## 2 Experimental design, materials and methods

**Fig. 1:**
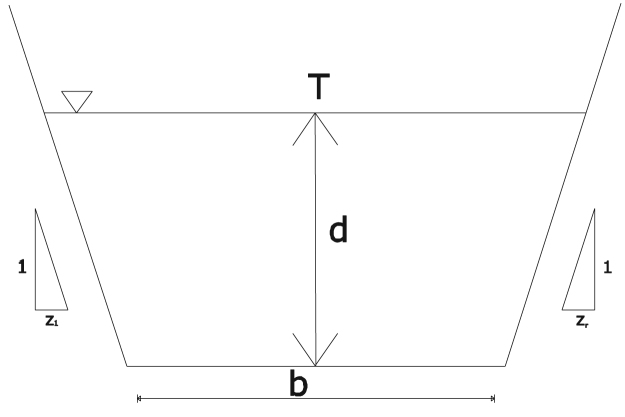
Cross-section of the basin to calculate other parameters.

**Fig. 2:**
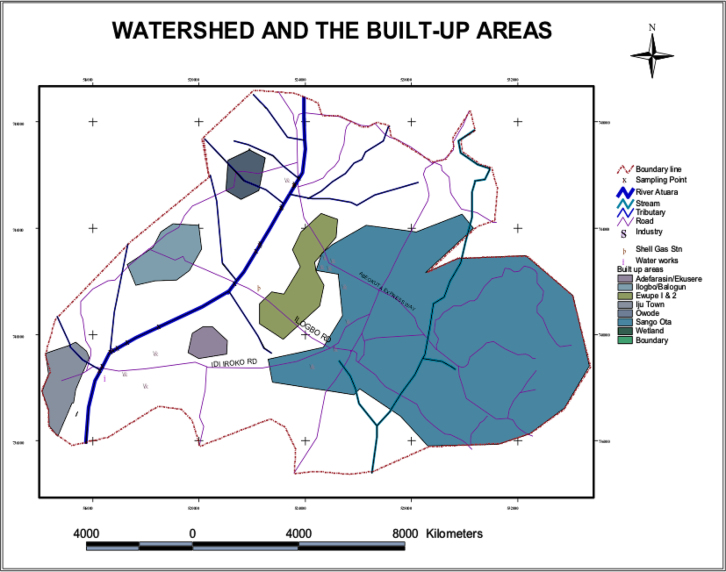
River Atuwara and the built-up areas.

Hydro-geometric data (such as depth, width and side slopes) of the Atuwara River were collected along Sixteen referenced points. The Sixteen referenced points (which is perpendicular to the direction of the river flow) were taken with the use of a boat and a Speedtech portable depth sounder. A global positioning system (GPS) unit was used to get the location of the sixteen-referenced point within Atuwara river. Fig. 2 shows the River Atuwara Watershed and built-up areas, while Fig. 1 is a plot of cross-section within the Atuwara river system, and their respective hydro-geometric channel label.

A digital elevation model (DEM) through the use of GPS is used to derive slope, slope length, aspect and other related parameters. The GPS, a global positioning system (Garmin GPS map 76) is navigating equipment. It is a small hand held receivable used to provide global positioning information (accurate to within 10–20 m). It is a cheap, flexible, convenient and relatively accurate device used to determine the position of people and devices naming anywhere around the globe. Values from Chow (1959) were used to estimate the Manning's roughness coefficient. The oxygen reaeration was gotten through Eq. (1) (O’Connor-Dobbins Formula) [6].

(1) $$K_{a}\;=\;3.93\frac{U^{0.5}}{H^{1.5}}$$

*K_a_*=Oxygen reaeration, *H*=Depth (m), *U*=Velocity (m/s).

The dispersion was analysed as the function of Eq. (2) [8]. Where *D*=Dispersion (L^2^/T).

*d* = depth or stage (L)

(2) $$D\;=\;\frac{0.01V^{2}W^{2}}{dU^{*}}$$

*g*=acceleration due to gravity (L/T^2^)=9.8 /m/s^2^, *s*=slope (L/L) (channel slope), *W*=width (L) $U^{*}=\sqrt{\mathrm{gHS}}$, *H*=mean depth or (*d*)

### 2.1 Study area

River Atuwara, located in Ado-Odo/Otta local government with co-ordinates 523883N 745372E in Ogun state. River Atuwara moves transversely toward other neighboring villages and serve as a water source [9], [10]. Fig. 2 shows the river and other built-up areas. The course of River Atuwara flows westward toward the Atlantic Ocean.

### 2.2 Data collection and processing

**Fig. 3:**
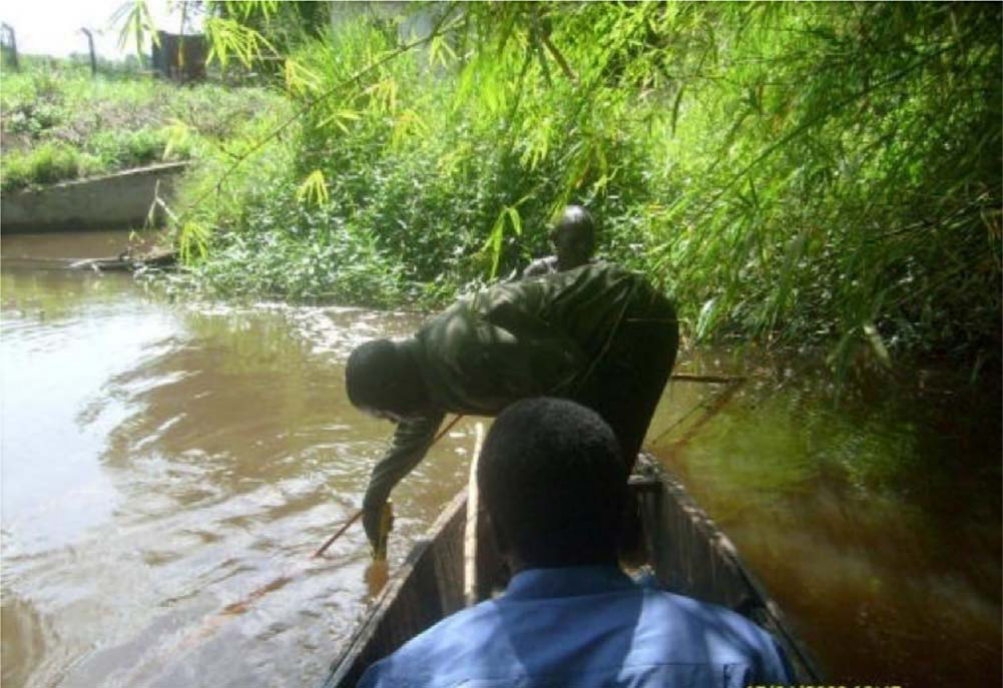
Hydro-geometric measurement on River Atuwara.

After collecting the hydro-geometric cross-sectional data, the hydro-geometric data was analyzed with the use of Microsoft office (Excel). The study assumes that an irregular channel cross-sections can be represented with hydraulically equivalent (that is, area to wetted perimeter remains the same) trapezoidal cross-sections as shown in Fig. 1. The hydro-geometric data was processed to determine the average depth of each cross-section, assuming the top-width of each cross-section were unchanged. Methods and processes of measurements, data collection and recordings employed along the river course are shown in Fig. 3.

### 2.3 Statistical analyses

**Fig. 4:**
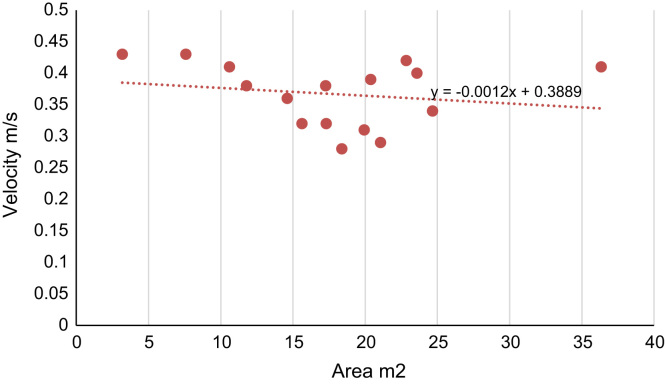
Velocity of River Atuwara against the area.

**Fig. 5:**
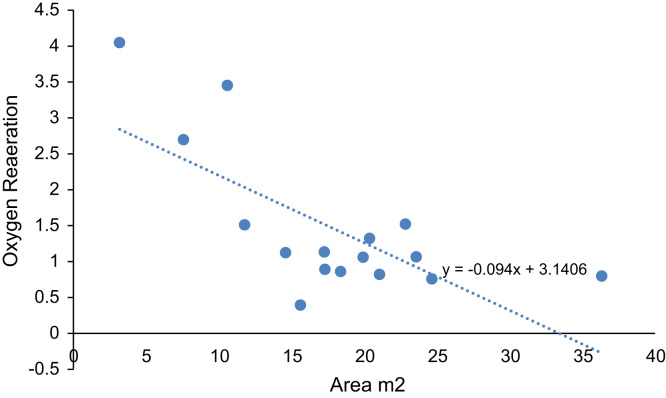
Oxygen reaeration of River Atuwara against the area.

**Fig. 6:**
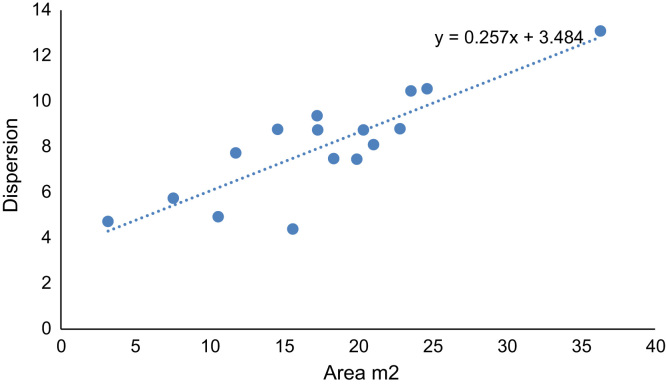
Dispersion against the area of River Atuwara.

The statistics analyses such as comparison of various unit of measurements are applied. The statistical summaries are shown in Fig. 4, Fig. 5, Fig. 6. The relationship between two-compared variable can obtained through the coefficient of the x-variable (gradient) in the regression equation indicated in Fig. 4, Fig. 5, Fig. 6. Negative gradient indicates inverse relationship while positive gradient shows direct relationships.

## Funding

The authors received no direct funding for this research.
