# Supplementary material for: Hydro-geometrical data analyses of River Atuwara at Ado-Odo/Otta, Ogun State
Source: Data Brief. 2018 Apr 25;18:1795–801. doi: 10.1016/j.dib.2018.04.071 (PMC5998175; doi:10.1016/j.dib.2018.04.071)
Supplement: Supplementary file 1 — Supplementary material [file mmc1.docx]

| Data accessibility | *All the data are present in the data article and continuous monthly data as supplementary materials.* |
| --- | --- |

**Value of the data**

The hydro-geometric data presented are suggestive for the following purposes;

- The data can be used to develop some numerical models that simulate and predict the transport and fate of organic pollutants in the environment (1–7).
- The dataset helps to describe the temporal and spatial behavior of pollutants and nutrients in the Atuwara River.
- These field observatory data can be used to validate predictive model for various hydrological seasons.
- The hydro-geometric data set can serve as an indicator to decision makers for consideration of current and futuristic water pollution controls.

# Data

*The dataset comprises of hydro-geometric analyses of selected sampling points on the River Atuwara, located in Ado-Odo/Otta, in southwest Nigeria. The hydro-geometric data was collected with the use of equipment such as depth meter, paddled boat, tape measure, and a global positioning system. Figure 3 is illustrative of the hydro-geometric data collection process. Geometric values are shown in Table 1, with their respective unit standards. Relationships between various units of measurement were derived statistically and presented in Figures 4-6.*
